# Supplementary material for: Evidence supported by Mendelian randomization: impact on inflammatory factors in knee osteoarthritis
Source: Front Med (Lausanne). 2024 May 28;11:1382836. doi: 10.3389/fmed.2024.1382836 (PMC11165061; doi:10.3389/fmed.2024.1382836)
Supplement: Supplementary file 1 [file Data_Sheet_1.ZIP › data/GCST90274798_5e6/798.forest.pdf]

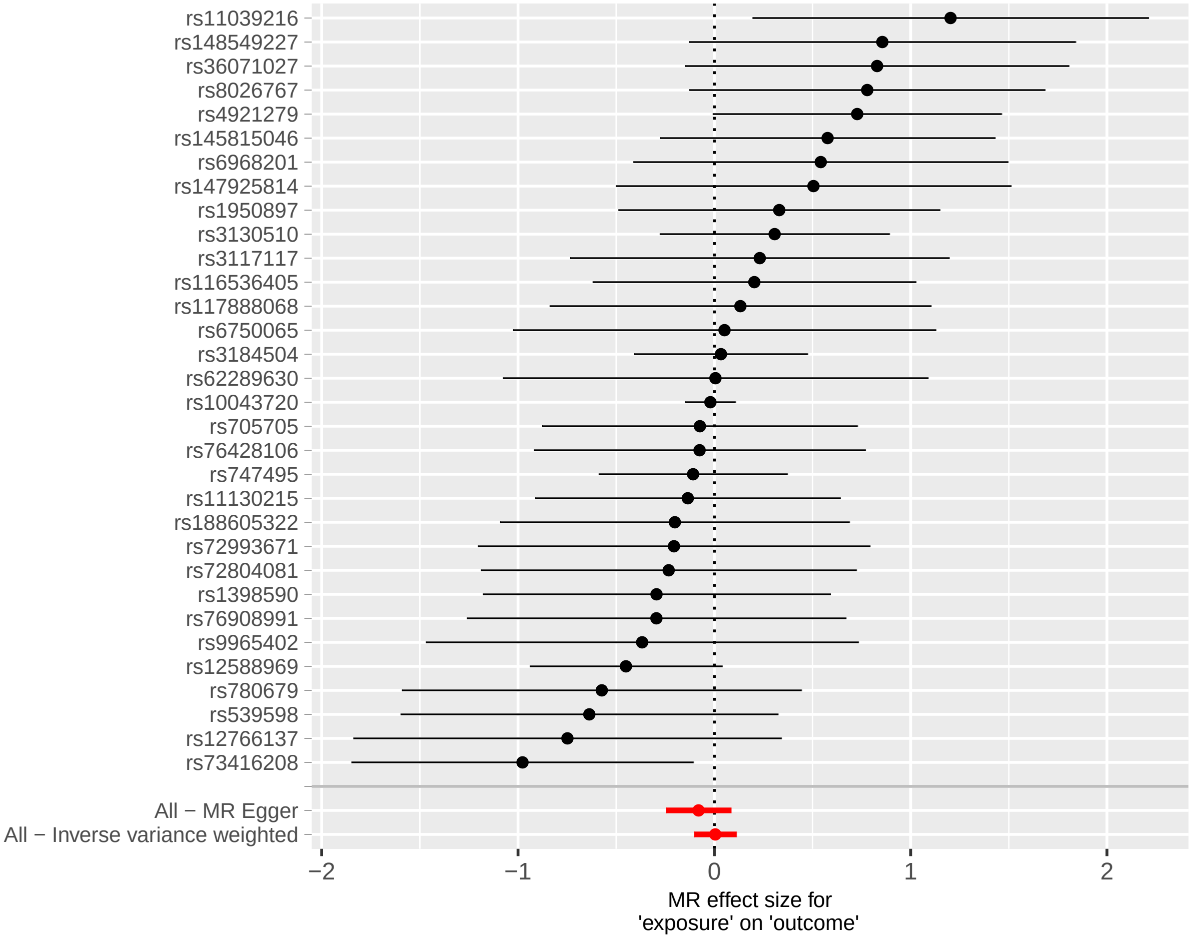
rs11039216
rs148549227
rs36071027
rs8026767
rs4921279
rs145815046
rs6968201
rs147925814
rs1950897
rs3130510
rs3117117
rs116536405
rs117888068
rs6750065
rs3184504
rs62289630
rs10043720
rs705705
rs76428106
rs747495
rs11130215
rs188605322
rs72993671
rs72804081
rs1398590
rs76908991
rs9965402
rs12588969
rs780679
rs539598
rs12766137
rs73416208
All − MR Egger
All − Inverse variance weighted
−2
−1
0
1
2
MR effect size for
'exposure' on 'outcome'
